# Are lip prints hereditary? A systematic review

**DOI:** 10.1007/s00414-023-02987-2

**Published:** 2023-04-03

**Authors:** Tânia Chaves, Álvaro Azevedo, Inês Morais Caldas

**Affiliations:** 1 Departamento de Ciências da Saúde Pública E Forenses E Educação Médica, Faculdade de Medicina, Universidade Do Porto, Porto, Portugal; 2 Faculdade de Medicina Dentária da Universidade Do Porto, Porto, Portugal; 3 Epidemiology Research Unit (EPIUnit), Institute of Public Health, University of Porto, Porto, Portugal; 4 Laboratory for Integrative and Translational Research in Population Health (ITR), Porto, Portugal; 5 TOXRUN–Toxicology Research Unit, University Institute of Health Sciences, Gandra, Portugal; 6 Department of Life Sciences, Centre for Functional Ecology (CFE), University of Coimbra, Coimbra, Portugal

**Keywords:** Forensic science, Criminal investigation, Human identification, Cheiloscopy, Lip prints, Heredity

## Abstract

Hereditary lip prints have been studied by several researchers. However, the literature shows no consensus among the scientific community regarding this topic. Therefore, the aim of this study was to conduct a systematic review to gather evidence to clarify whether the surface structure of lip prints is hereditary and, consequently, if a familial relationship between individuals can be established through the analysis of lip prints. The systematic review was performed following the PRISMA (Preferred Reporting Items for Systematic reviews and Meta-Analyses) guidelines. A bibliographic survey was conducted in PubMed, Scopus, and Web of Science databases, restricted to articles published between 2010 and 2020. Studies were selected according to eligibility criteria, and then the study data were collected. The risk of bias of each study was assessed and applied as additional inclusion or exclusion criteria. The results of the articles eligible for analysis were synthesized by a descriptive approach. In the seven included studies, methodological variations, including the definition of similarity, that contribute to the heterogeneity of results were identified. The data gathered allowed to conclude that there is no strong scientific evidence to support the hypothesis of the existence of heredity in the surface structure of lip prints, since it was not proven that similarities between parents and children occur systematically in all families.

**Supplementary Information:**

The online version contains supplementary material available at 10.1007/s00414-023-02987-2.

## Introduction

The red area of the lips, also called Klein’s zone [1, 2], is covered by several lines, the furrows and lip wrinkles, which vary in number, thickness, length, ramification and position [3]. The combination of these variations gives each individual a unique lip pattern [1]. When in contact with a surface, lips produce a particular mark: the lip print [4]. Lip prints can be found on glasses, paper napkins, certain foods, clothing, photographs, cigarette butts, glass and mirrors, tape in situations where the victim is gagged, on human skin, and open airbags, among others [5–8].

The uniqueness of lip prints allows to perform a comparative identification between a lip print found at a crime scene and the suspect’s print to confirm if there is a match [9]. However, when this is not possible mainly due to the lack of suspects, lip prints may help to estimate other relevant features [10–12], and some researchers think they can help to establish a family relationship between two or more lip prints left at the crime scene. This is only possible if the surface structure of lip prints is hereditary, that is, if lip prints present similarities between family members. The heredity of the surface structure of lip prints has been studied by several researchers [13–16], but so far, no consensus among the scientific community was reached. The need for more reliable answers from which investigators can draw conclusions and make decisions about the heredity of lip prints is evident. Therefore, the aim of this study was to conduct a systematic review to assemble evidence and identify possible gaps concerning the heredity of the surface structure of lip prints.

## Material and methods

This systematic review was performed according to the Preferred Reporting Items for Systematic Reviews and Meta-Analysis (PRISMA) guidelines [17], and the protocol was registered in International Prospective Register of Systematic Reviews (PROSPERO) with the registration number CRD42022377108.

According to the patient/population, intervention, comparison, outcome (PICO) framework, the following research question was defined: “Is it possible to establish a familial relationship (outcome) between individuals (population) from the analysis of their lip prints (intervention)?” From this, a more specific review question was defined: “Is the surface structure of lip prints hereditary?”.

The bibliographic search was performed in the PubMed, Scopus, and the Web of Science Core Collection databases. The search query “((cheiloscopy OR “lip prints”) AND forensic)” present in the title, abstract, or keywords was used. This broad search query was intentional, with the objective of not missing any relevant information. The publication date was restricted to the last 10 years from the day the search was conducted, i.e., between October 23, 2010, and October 23, 2020, written in English, Portuguese, and Spanish. Table 1 shows the search strategy applied. All references obtained were entered and organized in the EndNote X9.3.3 reference management software.

**Table 1:** Search strategy applied in each database

PubMed
(("cheiloscopy"[Title/Abstract] OR "lip prints"[Title/Abstract]) AND "forensic"[Title/Abstract]) AND ((2010/10/23:2020/10/23[pdat]) AND (english[Filter] OR portuguese[Filter] OR spanish[Filter]))
Scopus
TITLE-ABS-KEY (( cheiloscopy OR "lip prints") AND forensic) AND PUBYEAR > 2009 AND ( LIMIT-TO ( LANGUAGE, "English") OR LIMIT-TO ( LANGUAGE, "Portuguese") OR LIMIT-TO ( LANGUAGE, "Spanish"))
Web of Science Core Collection
(cheiloscopy OR “lip prints”) AND forensic (topic) Refined By: Languages: English Timespan: 2010–10-23 to 2020–10-23 (Publication Date)

After the bibliographic search was performed, duplicate references were removed. Next, the studies were assessed for eligibility according to predefined criteria (Table 2). All the review studies detected during the bibliographic search or in the reference management software were removed. Then, the remaining studies were assessed for eligibility by reading the title, abstract, and full text. The studies’ selection was carried out independently by the three authors. Whenever there was disagreement between the reviewers regarding the eligibility of a study, it was considered enough for one reviewer to consider the study eligible to move it on to the next stage.

**Table 2:** Inclusion and exclusion criteria

Inclusion criteria
a) Studies assessing whether lip prints are hereditary
Exclusion criteria
a) Studies that do not investigate the heredity of lip prints b) Reviews c) Abstract not available

After the selection process, the following data were extracted from the included studies: authors and year, aims, sample size (including number of male and female participants), age group, population, lip print collection method, analysis instrument, classification used, lip area analyzed, statistical analysis method, and results. Data were extracted by a reviewer and confirmed by a second reviewer. Any disagreement was resolved by consensus**.**

To assess the risk of bias, a list of criteria was developed based on the Critical Appraisal Checklist for Analytical Cross Sectional Studies from the Joanna Briggs Institute (JBI) [18]. The list was composed of 10 different domains (Table 3). For each domain, a maximum of five answer possibilities were applied: “Yes,” “Not totally,” “No,” “Not reported,” and “Not applicable.” The risk of bias low/null, medium, high, and uncertain was assigned to those domains answered with “Yes,” “Not totally,” “No,” and “Not reported,” respectively. The three authors were involved in this process. The risk of bias was applied as an additional inclusion or exclusion criterion. In this process, we only considered the risk of bias in the “Statistical analysis” and “Results presentation” domains, because these are domains with great relevance in the internal validity of the study. In this sense, whenever an article presented a high risk of bias in at least one of these two domains, it was excluded (Table 4).

**Table 3:** Risk of bias assessment criteria

1. Is the aim well defined?
2. Are the characteristics of the study population clearly specified?
3. Are the inclusion or exclusion criteria of participants specified?
4. Is the methodology presented and appropriate?
5. Was intra-rater reliability assessed?
6. Was inter-rater reliability assessed?
7. Was the statistical analysis applied adequate and well explained?
8. Is there an explicit and error-free results presentation?
9. Does it answer the study aim?
10. Are the conclusions based on the study results?

**Table 4:** Studies excluded due to risk of bias and their analysis

Authors and year	Definition of the aim	Description of the population data	Presentation of the inclusion or exclusion criteria of the participants	Presentation of the methodology	Assessment of reliability		Statistical analysis	Presentation of results	Answer to the aim	Rationale of the conclusions
					Intra-rater	Inter-rater				
Rekha et al. 2015	↓	→	↓	↓	•	•	↑	→	↓	↓
Miglani et al. 2016	↓	→	↓	↓	•	•	→	↑	↓	↓
Mala et al. 2017	↓	↓	↓	→	•	•	↑	↓	↓	↓

•, Uncertain risk of bias;↑, high risk of bias; → , medium risk of bias;↓, low/null risk of bias

The results were synthesized using a descriptive approach (Table 5). A quantitative synthesis, such as meta-analysis, was not performed due to the heterogeneity of lip print collection techniques, lip areas analyzed, definitions of similarity adopted, and statistical methods applied among the studies.

**Table 5:**
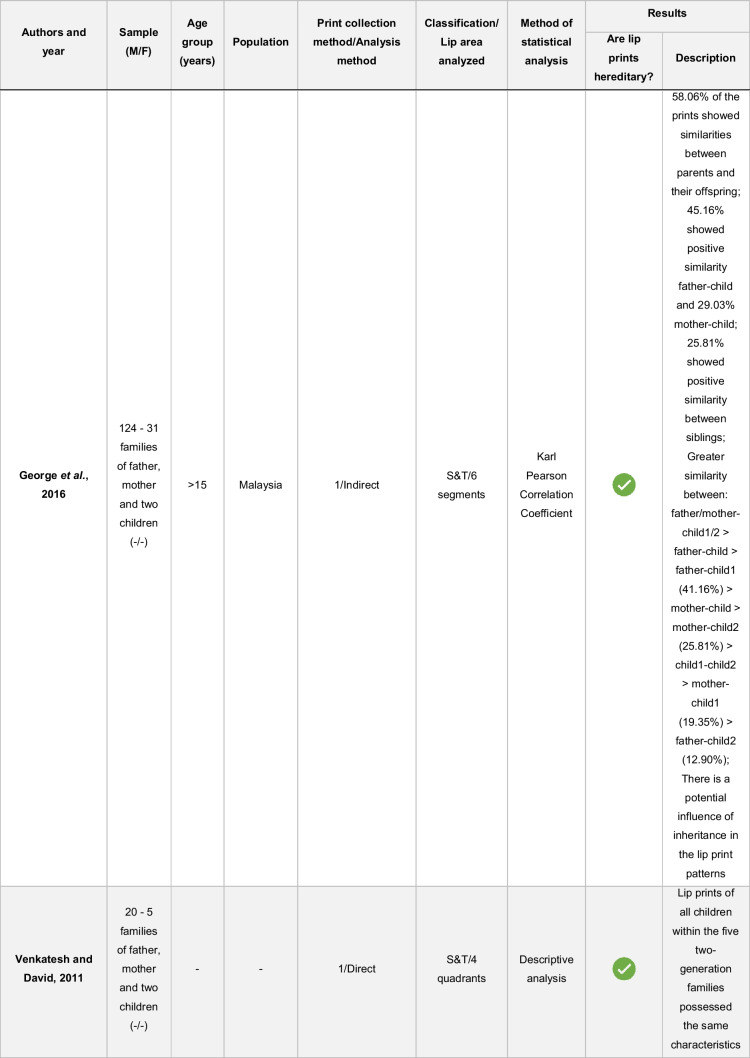

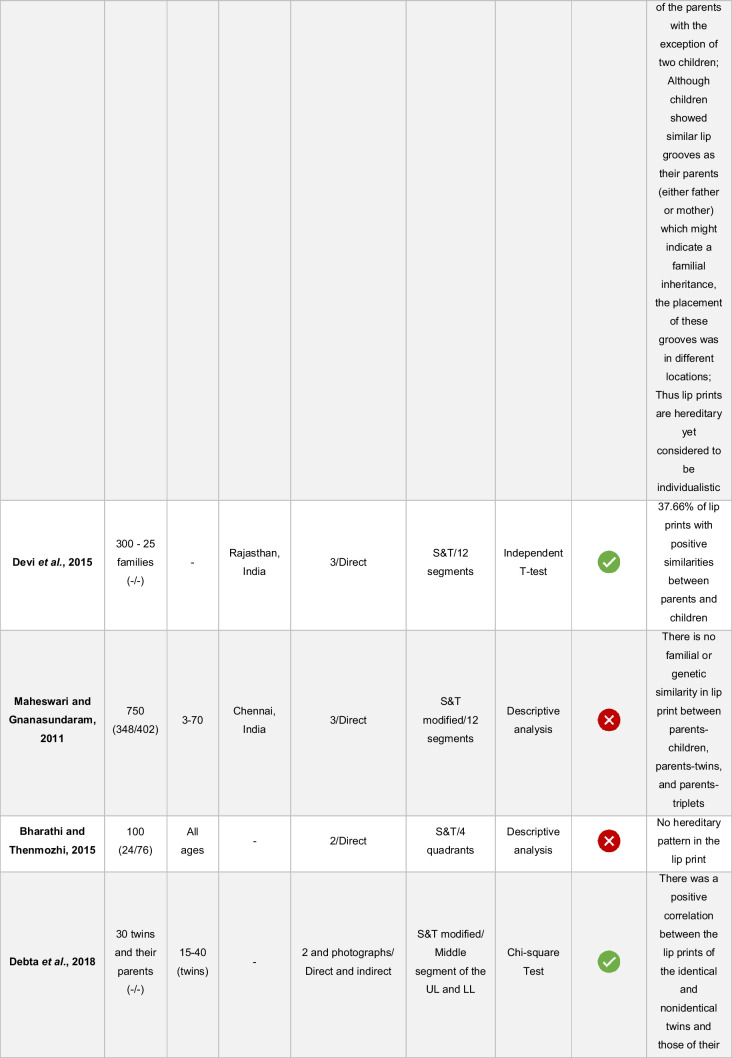

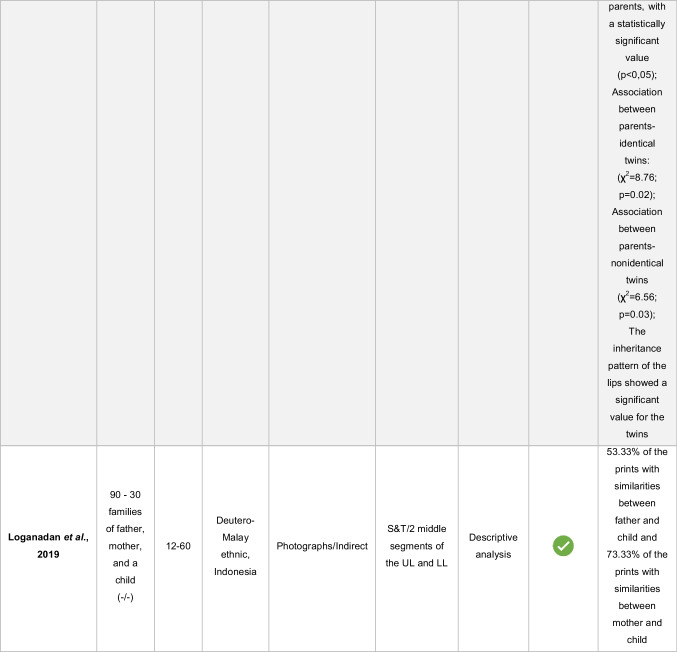
Distribution of variables of the included articles

M/F: male/female; UL – upper lip; LL, lower lip

“- “: the variable was not reported by the study authors

-Yes.  -No

## Results

**Fig. 1:**
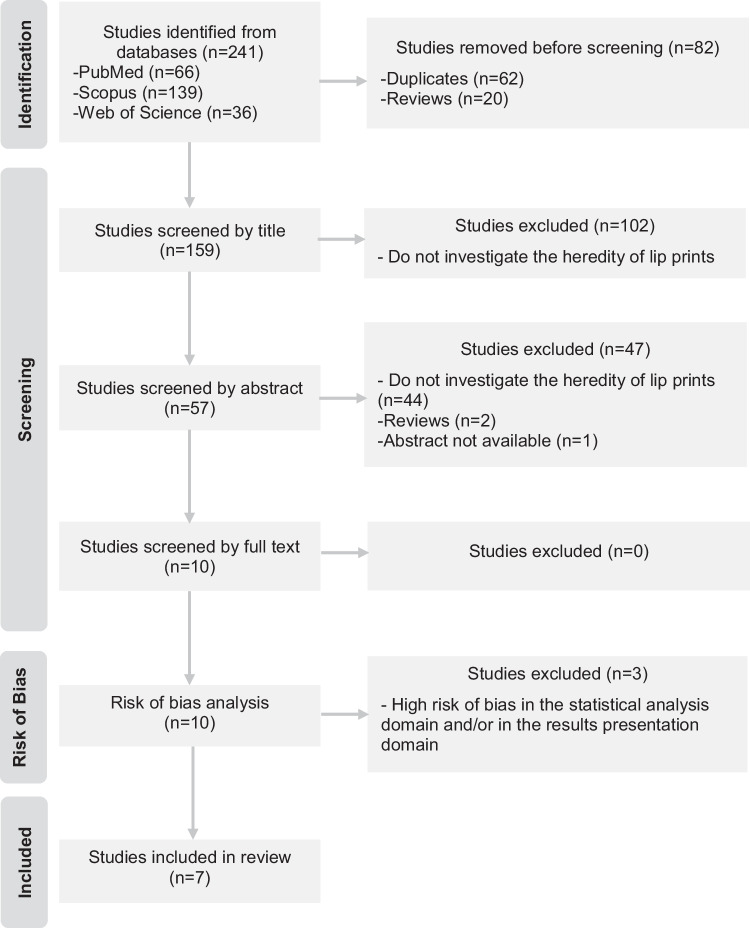
Flow diagram representing the selection of studies

As recommended by the PRISMA guidelines, the selection of studies was documented in detail in a flow diagram (Fig. 1). The search strategy identified a total of 241 studies. After removing duplicate studies, 169 articles were excluded by applying the eligibility criteria, and three articles were excluded in the risk of bias analysis. Thus, seven studies were included in this systematic review.

### Collection and analysis of lip prints

The procedure employed during the collection and analysis of lip prints varied significantly between studies. Regarding the method applied to collect the participants’ lip prints, it was possible to identify four different collection methods (Table 6).

**Table 6:** Lip print collection methods applied by the different studies

Method 1	• Lipstick application on the lips • Application of cellophane tape on the lips in order to register the lip print • The cellophane tape is pasted on paper
Method 2	• Lipstick application on the lips • Participants are asked to rub their lips in order to spread the lipstick evenly • Application of cellophane tape on the lips in order to register the lip print • The cellophane tape is pasted on paper
Method 3	• Lipstick application on the lips • The lip print is recorded directly on paper
Photographs	• Photographs are taken directly to the participants’ lips

In lip print analysis, the researchers used two types of instruments: magnifying lens which include the magnifying glass or the stereomicroscope (hereinafter referred to as the direct method) and image editing software such as Adobe Photoshop (hereinafter referred to as the indirect method).

To analyze and classify the labial grooves, several authors have chosen the Suzuki and Tsuchihashi (S&T) classification [19]. However, in some studies, this classification was used with alterations, including the addition of type I′ to type I and the omission of type I′.

**Fig. 2:**
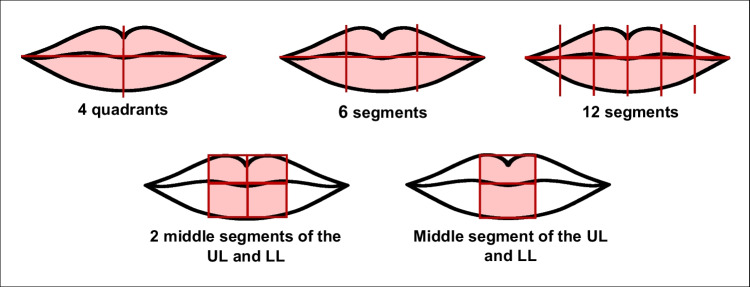
Illustration of the different lip zones analyzed by the studies. “UL” means “upper lip” and “LL” means “lower lip”

A pronounced heterogeneity was observed in the analyzed lip area. The choices included analyzing the whole lip divided into four, six, or twelve segments. The area to be analyzed was also limited to more restricted zones (Fig. 2).

### Characteristics of eligible studies

The sample in the different studies ranged from 20 to 750 participants, from all age groups, and from Malaysia, India, and Deutero-Malay ethnic. Three studies do not mention the population of origin of the volunteers (Table 5).

**Fig. 3:**
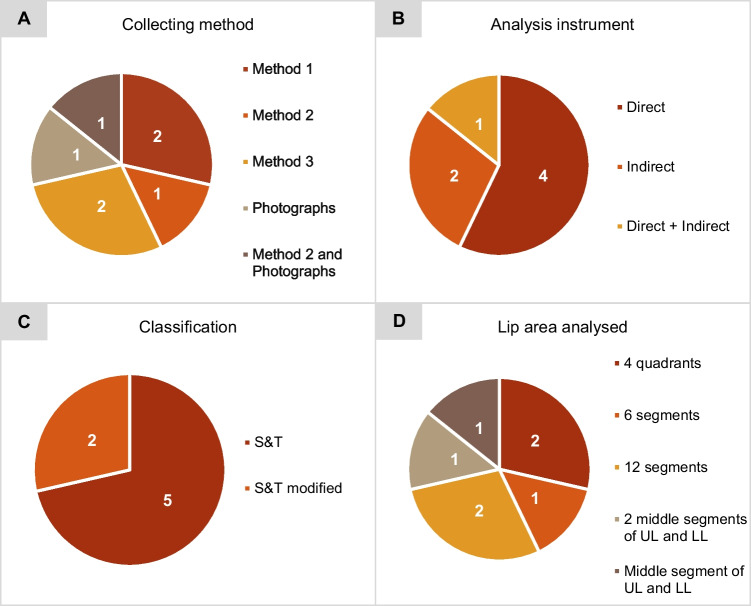
Distribution of lip print collection and analysis techniques among the studies: **A** lip print collection method; **B** analysis instrument, **C** classification, and **D** lip area analyzed. “UL” means “upper lip” and “LL” means “lower lip”

In the lip print collection, methods 1(consisting of lipstick application on the lips, application of cellophane tape on the lips to register the lip print, and pasting the cellophane tape on paper) and 3 (consisting of lipstick application on the lips, the lip print is recorded directly on paper) were the most applied methods. The direct method of lip print analysis was used in more than half of the studies (4/7), as well as the S&T classification (5/7). Regarding the area of the lip considered for analysis, in five studies, the authors analyzed the whole lip, divided into quadrants, six segments or twelve. In two studies, a smaller area of the lip was analyzed (Fig. 3).

### Risk of bias

**Fig. 4:**
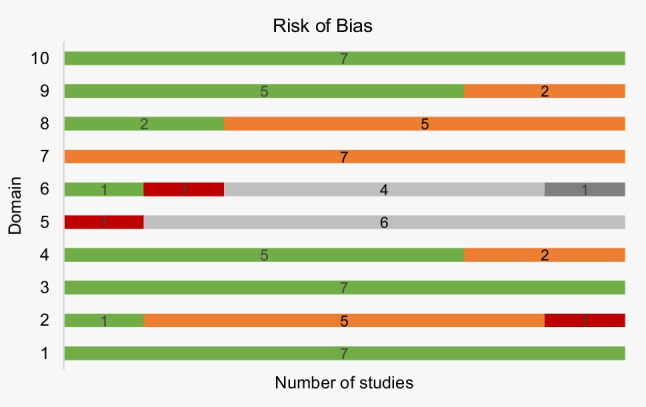
Frequency of studies by level of risk of bias and for each domain

**Fig. 5:**
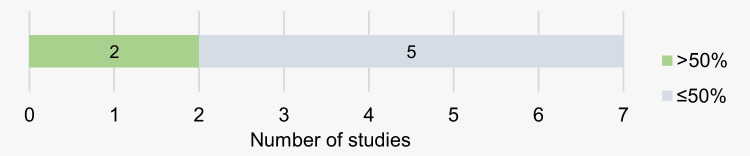
Frequency of studies with more and less than 50% of domains with low/null risk

The risk of bias is presented in detail in Supplementary Table 3 and Fig. 4. In domains 1, 3, and 10, all articles (7/7) were rated as low risk. In population characterization, most studies (5/7) had a medium bias risk, as only part of the population features was described. In the study by Venkatesh and David [5], the highest bias risk was assigned, because only one characteristic of the participants was mentioned. In the domain referring to methodology, the use of the modified S&T classification, considered not fully valid, contributed to the attribution of the medium bias risk in two studies [15, 20]. In domains 5 and 6, most articles did not mention if any strategy was used to evaluate inter- and intra-rater reliability. This was only described in only one study [13], but the methodology used is arguable, resulting in a high bias risk. As for the statistical analysis, all studies (7/7) had a medium bias risk, because they did not meet the assumptions for their realization or an alternative analysis would have been more appropriate. Regarding domain 8, in most of the articles (5/7), the lack of measures or tables to prove the results described in the text justified the classification with medium risk. In domain 9, low risk of bias prevailed. Overall, only two studies achieved low/null risk of bias in more than half of the domains (Fig. 5).

### Results of the selected studies

The hypothesis of the existence of heredity in the surface structure of lip prints was proven in five studies and refuted in two (Table 5). Although five articles agreed on the existence of similarities between the lip patterns of parents and their children, it is also possible to verify that this was not observed in all sampled individuals. In the article ranked highest in the analysis of bias risk [14], the authors identified similarities between parents and children in 58.06% of the lip prints, which leads to the inference that the same was not observed in 41.94% of the prints analyzed. Similarly, in the article ranked immediately below [5], five children had the same characteristics as their parents except for two. The authors add that although the children had similar lip grooves to their parents, they were in different locations, which led them to believe that even though they are unique, lip prints are hereditary. In the same sequence of articles, the research developed by Devi et al. proved that 37.66% of lip prints showed similarities between parents and children, while 62.34% did not [21]. At the lowest level of bias risk are the last two studies. In the study by Debta et al., a statistically significant association was found between lip prints of monozygotic twins and those of their parents (*p* = 0.02) as well as between lip prints of dizygotic twins and those of their parents (*p* = 0.03), indicating the existence of heredity according to the authors [20]. In the study by Loganadan et al., 53.33% of the children’s prints showed similarities to their fathers’ lip prints and 73.33% to their mothers’ prints. Again, similarities between parents and sons were not observed in all subjects [13]. The presence of more similarities between mothers and children than between fathers and children does not coincide with the results of George et al., who indicate just the opposite: there are more similarities between fathers and children than between mothers and children. In addition to the similarities found between fathers and sons, these researchers also found that 25.81% of lip prints showed similarities between siblings [14].

In the study by Maheswari and Gnanasundaram, it was proven that there was no familial or genetic similarity in lip print between parents and children, parents and twins, and between parents and triplets [15]. Bharathi and Thenmozhi also confirmed that there are no heritable lip patterns [16].

## Discussion

From the included studies, it is possible to verify that there are several inconsistencies regarding the existence of heredity in lip prints, as five studies have shown that lip prints are hereditary and two studies have shown the opposite. One of these two studies does not present any type of evidence that could justify the statement “There is no familial or genetic similarity in lip print between parents-children, parents-twins, and parents-triplets” [15], which makes a critical interpretation impossible. In the other study, the authors conclude that there are no hereditary patterns from the premise “No two lip prints were matched with each other” [16]. However, the existence of different lip prints does not necessarily mean that they are not hereditary. Of course, the parents’ lip prints will have to be different from those of their children due to genetic variability. Still, some similarities may exist that do not interfere with the character of uniqueness. On this matter, Tsuchiachi [22], in 1974, observed that in identical twins, although lip print patterns were duplicate, in detail, they were not exactly the same. In fact, although the lip print pattern in each pair of twins were neatly the same, at close inspection, they were not identical. Regarding the lip print patterns’ similarities between children and their families, Tsuchiachi [22] referred that the patterns were extremely similar but not the same. Similar results were obtained by Domiaty et al. [23], referring that although monozygotic twins showed some similarity, the lip-print patterns were not identical.

Thus, the authors’ reasoning [15, 16] leaves the reader in doubt, as they may not have paid attention to small similarities between parents and children that, hypothetically, would be indicators of heredity. This position agrees with previous and more ancient research, such as the one from Hirth et al. [24] who state that a genetical basis of lip prints pattern does, in fact, exist.

In the studies that demonstrated that the surface structure of lip prints is hereditary, similarities were not observed between all the lip prints of parents and children; besides, the percentage of similarities found is different from study to study. In this regard, it is important to define what is meant by “similarity,” or rather, what each study considered a “positive similarity.” In the study by George et al., the similarity between two lip prints was considered positive when more than three segments had the same pattern [14]. However, the authors do not clarify whether it would be the dominant pattern of each segment and whether the exact location of the furrows was considered. Venkatesh and David, on the other hand, considered the prints to be similar when they had the same types of grooves even if they were in different locations [5]. That is, unlike the previous paper in which the grooves had to be the consistent in the same segments, these authors did not take their location into consideration. Loganadan et al. assumed similarity when the prints of father or mother and child had at least two same types of grooves even if located in different segments [13]. In another research from Domiaty et al. [23], not included in the present systematic revision for date issues, they also refer to this concept of similarity/ dissimilarity, not explaining, however, what these concepts exactly mean. Thus, what for some authors would have been assessed as a positive similarity, for others, was not. Now, the various interpretations of “similarity” explain by themselves the heterogeneity of the results obtained in the studies. In fact, this is an important point that needs to be well defined to avoid lack of external validity. To understand its relevance, consider the following example: individual B, son of A, presents the same types of grooves as his father (types I, III, and IV) although in different locations. According to the definition of similarity adopted in the study by Venkatesh and David [5], if the prints of both were found at a crime scene, they would be identified as belonging to a father and his son since they are similar. However, it is very likely that an individual, randomly selected from the population, has the same types of grooves as individual A (the father) even if in different locations. Then, the hypothesis of heredity, according to this definition of similarity between parents and children, is weakened and, in practice, has no identifying value. In addition, the studies analyzed different lip areas, which also contributes to the variation in results, because while some researchers looked for similarities between family members across the whole lip print, others only looked for similarities in more restricted areas of the lip. Thus, it becomes necessary in studies with a larger sample size to analyze whether all father/mother and son pairs present similarities and what their typology is, namely, the configuration of the grooves, their location and frequency in the print, and the probability that one or more features may occur in the population. Only then will the conditions be created for a more correct definition of similarity indicating heredity.

Besides the different definitions of similarity adopted, it is worth noting the heterogeneity of the collection and analysis techniques applied, which may also have been determinant in obtaining different results, since the way these techniques are performed may affect the correct reading of the prints and, consequently, influence the results of each study [25–30].

Regarding collection, the method employed is a fundamental step to ensure the print quality. Recording the lip print is a technique-sensitive task and, therefore, depending on how the print is collected, its quality may vary. Thus, choosing the most appropriate method is essential to ensure the success of the analysis. Costa and Caldas [31] tested four methods and found that the application of lipstick without rubbing the lips followed by transfer to cellophane tape (method 1) is the method that provides the best lip print reading. This was, in fact, one of the collection methods most used by the articles included in the systematic review. A 2010 study [32] showed that the method 2 is the most appropriate because of the good quality of the print, low technical difficulty, and speed of the procedure. Regardless of the advantages they may present, the main limitation reported by studies in using the conventional methods is in the amount of lipstick applied that, in excess, can decrease the print quality [33]. Evidence shows that prints taken with a thinner layer of lipstick have better quality [34].

The pressure applied during collection and the direction can also alter the appearance of the prints [22] and consequently affect correct identification. Human lips are naturally mobile [29]; therefore, the pattern of lip wrinkles depends on how the muscle relaxes to produce the print [1]. To overcome this limitation and to avoid errors in the analysis, some researchers have used the photography technique to record lip prints instead of the traditional lipstick and paper recording method [13, 20]. Photography has been suggested as the most appropriate method for taking lip prints [20, 35]. To do so, it is very important to create good lighting conditions, as mismatched shadow and light areas may influence the quality of the images [3].

Regarding analysis, the direct method was the most used essentially because it is very practical and simple. On the other hand, it may not offer the best visualization of the prints. As for the indirect method, image editing software allows improving the visualization of prints by adjusting brightness, color, or contrast or enlarging details [26, 36]. In this way, the same lip print, with some imperceptible or overlapping details, may see its quality improved with the use of image editing software, while the simple use of the magnifying lens would not allow it. Thus, the analysis method applied may also influence the results, since better visualization of the prints will certainly lead to an increase in correct analyses.

Fingerprints and lip prints are frequently discussed together, as they are thought to share the same main principles (singularity, stability, and permanence) [10]. Regarding fingerprint hereditariness, some studies point to the existence of heritability in some dermatoglyphic characteristics (delta indexes and ridge counts for right hand, left hand, and both hands, and ridge counts for most individual fingers) and not in others (ridge counts) [37, 38]. So, an inheritable quality to fingerprints seems to exist. Pattern types are often genetically inherited, but the individual details that make a fingerprint unique are not [39].

The overall risk of bias weakens the validity of the results obtained, since most studies (5/7) failed to achieve low risk in more than half of the domains. The data collected from the best-ranked articles regarding the risk of bias highlights the existence of hereditary similarities among lip prints, which may be variable, depending mainly on the definition adopted. However, it should be noted that similarities between parents and children did not appear in all families, which suggests that this parameter may have limited relevance for criminalistics. Additionally, one could argue if similarities are enough or it is mandatory to reach a conclusion “without any reasonable doubts.” Page et al. [40] have discussed the need for singularity extensively, claiming that even though forensic scientists involved in fingerprint, firearms, toolmark analyses, and many more trust on the uniqueness proposition to back their statements regarding identification; this is not needed or even possible. Stoney [41] has stated more than 3 decades ago that trying to prove uniqueness using statistics was “a ridiculous goal.” So, more than driving for achieving this goal, impossible and unnecessary, more attention should be given to the way data is obtained. In regard to this, Page et al. [40] have stated “mistakes and misidentifications are not made because someone has an identical fingerprint to someone else in the world. They are made because of guesswork, poor performance, lack of standards, bias, and observer error.” Those were definitely some of the problems we have found performing this research.

## Conclusion

This systematic review identified methodological variations between studies, including variations in the adopted definition of “similarity” indicative of heredity, that contribute to the discrepancy in results and, therefore, to the lack of consensus regarding the existence of heredity in lip prints.

The data gathered allowed us to conclude that there is no strong scientific evidence to support the hypothesis of the existence of heredity in lip prints, since it was not proven that similarities between parents and children occur systematically in all families. Thus, establishing a familial relationship between individuals from the analysis of their lip prints left at the crime scene is an imperfect process.

## Supplementary Information

Below is the link to the electronic supplementary material.

Supplementary file1 (DOCX 25.3 KB)
