# Supplementary material for: Are lip prints hereditary? A systematic review
Source: Int J Legal Med. 2023 Apr 3;137(4):1203–14. doi: 10.1007/s00414-023-02987-2 (PMC10247594; doi:10.1007/s00414-023-02987-2)
Supplement: Supplementary file 1 — Supplementary file1 (DOCX 25.3 KB) [file 414_2023_2987_MOESM1_ESM.docx]

Supplementary table 3 - Risk of bias for each included study.

| Authors and year | Definition of the aim | Description of the population data | Presentation of the inclusion or exclusion criteria of the participants | Presentation of the methodology | Assessment of reliability | | Statistical analysis | Presentation of results | Answer to the aim | Rationale of the conclusions |
| --- | --- | --- | --- | --- | --- | --- | --- | --- | --- | --- |
|  |  |  |  |  | **Intra-rater** | **Inter-rater** |  |  |  |  |
| Maheswari and Gnanasundaram, 2011 (1) | ↓ | ↓ | ↓ | → | ● | ● | → | → | ↓ | ↓ |
| Venkatesh and David, 2011 (2) | ↓ | ↑ | ↓ | ↓ | ● | ● | → | ↓ | ↓ | ↓ |
| Bharathi and Thenmozhi, 2015 (3) | ↓ | → | ↓ | ↓ | ● | ● | → | → | ↓ | ↓ |
| Devi *et al.*, 2015 (4) | ↓ | → | ↓ | ↓ | ● | **NA** | → | → | → | ↓ |
| George *et al.*, 2016 (5) | ↓ | → | ↓ | ↓ | ● | ↓ | → | ↓ | ↓ | ↓ |
| Debta *et al.*, 2018 (6) | ↓ | → | ↓ | → | ● | ● | → | → | ↓ | ↓ |
| Loganadan *et al.*, 2019 (7) | ↓ | → | ↓ | ↓ | ↑ | ↑ | → | → | → | ↓ |

**NA -**  Not Applicable ● - Uncertain risk of bias ↑ - High risk of bias → - Medium risk of bias ↓ - Low/null risk of bias
